# Ocular involvement and visual outcome of herpes zoster ophthalmicus: review of 45 patients from Tunisia, North Africa

**DOI:** 10.1186/s12348-014-0025-9

**Published:** 2014-09-17

**Authors:** Rim Kahloun, Sonia Attia, Bechir Jelliti, Ahmed Zakaria Attia, Sana Khochtali, Salim Ben Yahia, Sonia Zaouali, Moncef Khairallah

**Affiliations:** 1 Department of Ophthalmology, Fattouma Bourguiba University Hospital, Faculty of Medicine, University of Monastir, Monastir, 5019, Tunisia

**Keywords:** Herpes zoster ophthalmicus, Varicella zoster virus, Uveitis, Keratouveitis, Keratitis, Visual outcome, Epidemiology

## Abstract

**Background:**

Ocular complications of herpes zoster ophthalmicus (HZO) may lead to substantial visual impairment. The purpose of this study was to characterize and analyze ocular involvement and visual outcome of HZO in patients from Tunisia, North Africa. This study is a retrospective chart review of 51 eyes of 45 patients with HZO.

**Results:**

Mean age was 44.5 years. Thirty patients (66.7%) were aged over 50 years. Twenty-four patients (53.3%) were male and 21 patients were female (46.7%). There was no statistically significant difference in gender distribution. Initial mean best corrected visual acuity (BCVA) was 20/50. Ocular manifestations included adnexal involvement (58.8%), keratitis (31.4%), keratouveitis (31.4%), isolated anterior uveitis (AU) (29.4%), intraocular pressure elevation (23.5%), oculomotor nerve palsy (5.8%), and optic neuritis (1.9%). Isolated AU (*p* < 0.001), isolated keratitis (*p* = 0.001), and intraocular pressure elevation (*p* = 0.013) were more likely to be concomitant to HZO active skin disease, while keratouveitis occurred more likely more than 1 month after HZO eruption (*p* < 0.001). AU and keratouveitis were more likely to be associated with age ≥ 50 years (*p* = 0.001 and *p* = 0.02, respectively). Ocular complications included neurotrophic keratopathy (1.9%), corneal opacity (5.9%), secondary glaucoma (7.8%), optic atrophy (1.9%), and postherpetic neuralgia (13.3%). Mean follow-up was 12 months. Mean final BCVA was 20/32; it was ≥ 20/40 in 78.4% of the eyes.

**Conclusions:**

Our study provided epidemiologic and clinical data of HZO in a Tunisian population. AU and keratitis were the most common ocular complications. Neurotrophic keratopathy was scarce. The overall visual outcome is good, with about three quarters of the treated patients maintaining VA of 20/40 or better.

## Background

Herpes zoster ophthalmicus (HZO) is a reactivation of the varicella zoster virus involving the ophthalmic division of cranial nerve V. Herpes zoster affects 20% to 30% of the population at some point in their lifetime, and approximately 10% to 20% of these individuals will have HZO [1]. Ocular involvement occurs in approximately 50% of HZO patients in the absence of prompt preventive antiviral therapy [1]. HZO is often associated with a chronic course characterized by significant ocular morbidity and may lead to significant visual impairment in the absence of prompt and adequate treatment. Data on HZO from North African region are lacking.

The purpose of the present study was to characterize and analyze ocular involvement and visual outcome of HZO in patients from Tunisia, North Africa.

## Methods

The charts of 45 consecutive patients (51 eyes) with HZO managed at the Department of Ophthalmology at Fattouma Bourguiba University Hospital of Monastir, Tunisia, from 1 January 2000 to 31 January 2012 were reviewed. The diagnosis of acute HZO was based on the presence of a primary vesiculomacular and dysesthetic skin rash within the ophthalmic dermatome. All patients were referred to our department from emergency department or by ophthalmologists. Patients were questioned concerning the history of the present illness, compromised immune status, medication use including immunosuppressive drug and topical or systemic antiviral therapy at presentation, renal failure, moderate or severe hypertension, diabetes mellitus, and previous relevant eye diseases such as chronic uveitis, keratitis, cataract, and glaucoma. All patients underwent detailed ophthalmic examination including adnexae examination, ocular motility, Snellen best corrected visual acuity (BCVA), slit-lamp examination, and fluorescein test. Adnexal involvement, including lid edema, blepharoconjunctivitis with macular rash involving the eyelids, conjunctival injection, vesicles, and petechial hemorrhages, was sought. Corneal sensitivity testing by comparison of a light touch with a cotton wool strand against the normal fellow eye was performed and recorded as normal, reduced, or absent. Grading of intraocular inflammation using standardization of uveitis nomenclature (SUN) working group criteria [2], tonometry, and dilated fundus examination with noncontact and contact lenses was also performed for all patients.

Ocular manifestations were defined as any ocular disease secondary to HZO. Intraocular pressure (IOP) elevation was defined as IOP ≥ 22 mmHg. The type of uveitis, onset, duration, and course was classified according to the SUN working group criteria [2]. Keratitis was divided into epithelial keratitis and stromal keratitis. When keratitis was associated to uveitis, we used the term keratouveitis.

Routine laboratory work-up including a complete blood count, erythrocyte sedimentation rate, C-reactive protein tests, and serum biochemical analysis was performed for all patients. Patients with bilateral involvement or aged less than 50 years underwent dermatologic and infectious diseases specialized examinations as well as serological examinations, including HIV antibodies and syphilis serology.

The presence or absence of postherpetic neuralgia (PHN) was also recorded. PHN was defined in our study as any symptom of pain or the use of pain medication for ocular symptoms documented in the medical record at least 3 months after onset of HZO [3].

Patients were divided into two groups: group I including patients with no ocular complications related to HZO and group II including patients with ocular complications related to HZO. Group II was also divided into two subgroups: group II_1_ including patients with ocular complications concomitant to HZO and group II_2_ including patients with ocular complications occurring at least 1 month after HZO (Table 1).

**Table 1:** Distribution of patients

**Groups**	**Number of eyes** ***N*** **= 51**	**%**
Group I: patients with no ocular complications related to HZO	4	7.8
Group II: patients with ocular complications related to HZO	47	92.2
Group II_1_: patients with ocular complications concomitant to HZO	32	62.7
Group II_2_: patients with ocular complications occurring at least 1 month after HZO	15	29.5

HZO, herpes zoster ophthalmicus.

All patients were treated with intravenous acyclovir 10 mg/kg 3 times daily or oral valacyclovir 3 g/day for 7 to 10 days. Topical corticosteroids, cycloplegics, topical antibiotics, topical beta-blockers, oral carbonic anhydrase inhibitors, and analgesics were prescribed when indicated. Patients with anterior uveitis (AU) received antiviral therapy for 8 to 14 weeks along with topical corticosteroid therapy tapering. The mean follow-up was 12 months (range 9 to 51).

Data entry and statistical analyses were performed using Windows-based SPSS statistical software. Proportions were compared between groups using the chi-square or Fisher's exact test, as appropriate. Association between variables was considered statistically significant for a confidence level of 95% (*p* ≤ 0.05).

## Results

Demographic data of our patients are given in Table 2. Mean age was 44.5 years (range 12 to 77). Thirty patients (66.7%) were aged over 50 years. There was no statistically significance in gender distribution in patients aged less or over 50 year old (*p* = 0.214) or in mean age between genders (*p* = 0.615). HZO was unilateral in 39 patients (86.7%). HZO affected the right eye in 21 patients (46.6%), the left eye in 18 patients (40%), and both eyes in 6 patients (13.3%). Associated systemic diseases included diabetes mellitus (9 patients; 20%), systemic hypertension (8 patients; 17.8%), and dyslipidemia (2 patients; 4.4%). Used medication included long-term corticosteroid therapy for back pain (1 patient; 2.2%) and immunosuppressive therapy for leukemia (1 patient; 2.2%) or for renal transplantation (1 patient; 2.2%). Serology testing revealed HIV infection in 2 patients (4.4%).

**Table 2:** Demographic characteristics and distribution of patients with herpes zoster ophthalmicus

	**Number of patients** ***N*** **= 45**	**%**
Age		
Mean age	44.5 years (range 12 to 77)	
Intervals		
<50	15	33.3
50 to 70	24	53.3
≥70	6	13.3
Gender		
Male	24	53.3
Female	21	43.7
Laterality		
Unilateral	39	86.7
Bilateral	6	13.3
Risk factors		
Diabetes	9	20
Immunosuppressive therapy	2	4.4
Long-term corticosteroid therapy	1	2.2
HIV infection	2	4.4

Of 6 patients with bilateral simultaneous HZO, 2 had diabetes, 2 were under immunosuppressive therapy and 1 had HIV infection, and the past medical history was unremarkable in the remaining patient.

Ocular symptoms included a red and painful eye in 42 patients (93.3%), blurred vision in 44 eyes (86.2%), and diplopia in 3 patients (6.7%). Initial BCVA ranged from 20/200 to 20/20 (mean 20/50). It was < 20/200 in 7 eyes (13.7%) and ≥ 20/40 in 15 eyes (29.4%). Group I included 4 eyes (7.8%) of 4 patients and group II 47 eyes (92.2%) of 41 patients, divided into group II_1_ (32 eyes; 62.7%) and group II_2_ (15 eyes; 29.5%) (Table 1).

Ocular manifestations included adnexal involvement (30 eyes; 58.8%), AU (31 eyes; 60.7%), keratitis with corneal hypoesthesia (16 eyes; 31.4%), oculomotor nerve palsy (3 eyes; 5.8%), and optic neuritis with optic disc edema (1 eye; 1.9%). No case of retinal involvement was recorded in our patients.

Adnexal involvement included lid edema (30 eyes; 58.8%), subconjunctival hemorrhage (23 eyes; 45.1%), and vesicular conjunctivitis (8 eyes; 25.8%).

Corneal hypoesthesia was recorded in 16 eyes (31.4%). Corneal involvement included stromal keratitis in 8 eyes (15.6%), epithelial keratitis in 5 eyes (9.8%), and concomitant superficial and stromal keratitis in 3 eyes (5.8%). Endotheliitis was not recorded in any affected eye.

Keratouveitis and isolated AU were observed respectively in 16 eyes (31.4%) and in 15 eyes (29.4%). AU was acute in 26 eyes (83.8%), recurrent in 2 eyes (6.4%), and chronic in 3 eyes (6.9%). Granulomatous keratic precipitates were found in 18 eyes (58.1%). Focal iris atrophy was recorded in 8 eyes (25.8%), Koeppe's nodules in 2 eyes (6.5%), pupillary distortion in 2 eyes (6.5%), and posterior synechiae in 5 eyes (16.1%). IOP elevation was noted in 12 eyes of 31 eyes with AU (23.5%) (Table 3).

**Table 3:** Ocular complications associated with herpes zoster ophthalmicus

	**Number of eyes** ***N*** **= 51**	**%**
Lid edema	30	58.8
Subconjunctival hemorrhage	23	45.1
Vesicular conjunctivitis	8	15.7
Corneal hypoesthesia	16	31.4
Keratitis	16	31.4
Epithelial keratitis	5	9.8
Stromal keratitis	8	15.7
Epithelial and stromal keratitis	3	5.9
Anterior uveitis only	15	29.4
Keratouveitis	16	31.4
Iris atrophy	8	15.7
Posterior synechiae	5	9.8
Pupillary distortion	2	3.9
IOP elevation	12	23.5
Glaucoma	4	7.8
Neurotrophic keratopathy	1	1.9
Oculomotor nerve palsy	3	5.9
Optic neuritis	1	1.9

IOP, intraocular pressure.

Oculomotor nerve palsy included sixth cranial nerve in 2 eyes (3.9%) and third cranial nerve in 1 eye (1.9%).

All patients received antiviral therapy 2 to 10 days (average of 4 days) after the onset of ocular symptoms. Keratitis gradually improved in all patients. There was neurotrophic keratopathy in 1 eye (1.9%) of a patient aged over 50 years and corneal opacity in 3 eyes (5.9%). IOP elevation was controlled with medical therapy in 8 (15.7%) of the 12 eyes with IOP elevation. Four eyes (7.8%) developed secondary glaucoma with optic disc cupping and visual field defects.

Oculomotor nerve palsy gradually resolved within few months in all 3 eyes initially involved. The case of optic neuritis developed optic disc atrophy with deep VA loss. Mean final BCVA was 20/32 (range 20/400 to 20/20). It was < 20/200 in 4 eyes (7.8%) and ≥ 20/40 in 40 eyes (78.4%).

Our statistical analysis showed that patients from group II_1_ are more likely to have isolated AU (*p* < 0.001), isolated keratitis (*p* = 0.001), and IOP elevation (*p* = 0.013) than patients with ocular complications occurring at least 1 month after HZO. However, keratouveitis occurred more likely in group II_2_ (*p* < 0.001). AU and keratouveitis were more likely to be associated with age ≥ 50 years (*p* = 0.001 and *p* = 0.02, respectively). There was no statistically significant relationship between ocular complications of HZO and gender (Table 4). PHN was recorded in 6 patients (13.3%).

**Table 4:** Correlations between ocular complications in herpes zoster ophthalmicus groups, age, and gender

**Ocular involvement**	**Group**			**Age (years)**			**Gender**			**Total**
	**II**_**1**_**%**	**II**_**2**_**%**	***p*** **value**	**≥50%**	**<50%**	***p*** **value**	**Female %**	**Male %**	***p*** **value**	
Anterior uveitis	38.3	27.66	0.27	45.10	15.69	0.001*	33.33	27.45	0.51	31
Isolated anterior uveitis	31.91	0	<0.001*	19.61	9.80	0.16	21.57	7.84	0.051	15
Keratouveitis	6.38	27.66	<0.001*	23.53	7.84	0.02*	17.65	13.73	0.58	16
Isolated keratitis	29.79	4.26	0.001*	15.69	15.69	1	11.76	19.61	0.27	16
IOP elevation	21.28	4.26	0.013*	15.69	7.84	0.21	9.80	13.73	0.53	12
Oculomotor nerve palsy	6.38	0	0.24	5.88	0	0.24	3.92	1.96	1	3
Iris atrophy	6.38	10.64	0.71	13.73	1.96	0.07	11.76	3.92	0.26	8

IOP, intraocular pressure. *statistically significant p values.

## Discussion

The estimates of herpes zoster ranged between 320 and 410 per 100,000 person-years [4]. A recent study from the Pacific region estimates the overall incidence rate of HZO at 30.9 per 100,000 person-years, which is approximately 10% of all estimates of herpes zoster overall [5].

In our study, 66.7% of patients were aged over 50 years, and 53.3% of patients were aged between 50 and 70 years. Previous studies showed that the incidence of HZO increases significantly with age [1],[5]. A recent case series reports that HZO affects similarly individuals aged younger than or older than 60 years, with the most common decade of onset between age 50 and 59 years [6]. In a recent study, the incidence rate for the subgroup of the population older than 65 years was approximately five times that of the rest of the population [5]. Increased age has been associated with a decrease in cell-mediated immunity, which is a crucial factor to avoid reactivation of the latent varicella zoster virus.

Ghaznawi et al. [6] showed that younger individuals were more likely to be healthy, with predisposing conditions such as immunosuppressive treatment, cancer, and HIV infection present in only 11.1% of patients who were younger at onset. In our study, immunosuppressive therapy and HIV infection were recorded in 3 (20%) of 15 patients aged less than 50 years.

The results of our study, consistent with data from a previous report [5], show no significant difference in HZO incidence between men and women overall and between specific age groups. However, some other studies found significant differences between incidence rates for men and women within specific age groups, but there was limited evidence of a significant difference overall between incidence rates for men and women [4],[7].

In the current study, a high incidence of ocular involvement due to HZO (92.2%) was found. This may be explained by a bias selection since, in our hospital, the majority of HZO cases without ocular complaints are usually referred to infectious disease specialists or dermatologists.

AU, with or without associated keratitis, was the most common ocular manifestation of HZO in our series, occurring in 60.8% of eyes, followed by epithelial and/or stromal keratitis recorded in 31.4% of eyes. Similarly, in a study including 73 immunocompetent patients from the Netherlands during a period of 6 months follow-up, AU was reported in 43.8% of eyes at initial examination, followed by stromal keratitis in 30.1% of eyes [8]. In another study, AU was recorded in 46.6% of cases and keratitis in 76.2% of cases [9]. In a series of 18 younger than 40 years old patients with HZO, AU was recorded in 50% of cases and stromal keratitis in 22% of cases [10]. In a recent study from the Pacific Islanders, a lower rate of AU was recorded (6% of cases); however, keratitis was recorded in 28.3% of cases [5]. In the latter study, all patients, with or without ocular involvement, were included; this may explain the lower proportion of AU.

In the current study, isolated AU and isolated keratitis were more likely to be concomitant to HZO eruption (*p* < 0.001 and *p* = 0.001, respectively); however, keratouveitis is more likely to occur at least 1 month after HZO (*p* < 0.001). A recent study shows the risk of AU increased significantly in the year following a diagnosis of HZO [11]. In addition, we found that both isolated AU and keratouveitis were more likely to occur in patients ≥ 50 years old (*p* = 0.001 and *p* = 0.02, respectively), and there was no statistically significant association between age and the occurrence of keratitis. In contrast, a recent study comparing the course of HZO in patients' younger vs. older than 60 years found that younger patients had more episodes of delayed pseudodendritiform keratitis and AU compared with older patients. However, prevalences of corneal perforation, corneal thinning, cataract formation, and glaucoma were similar between the two groups [6].

Despite the fact that about one third of our patients developed keratitis, only one patient aged over 50 years old developed neurotrophic keratopathy. This may be explained by the early antiviral therapy in our patients with an average of 4 days after the onset of HZO. While Ghaznawi et al. [6] found that neurotrophic keratopathy affected 19.6% of all patients and was more frequent in older patients. Similarly, Liesegang et al. [12] reported a 25% prevalence of neurotrophic keratitis.

In our series, oculomotor nerve palsy was recorded in 5.8% of eyes with complete resolution during follow-up. The reported incidence of extraocular muscle palsies in HZO ranged between 7% and 31% [13],[14], but many cases are asymptomatic and usually transient. The third oculomotor nerve is most commonly affected, followed by the abducens nerve and the trochlear nerve [15]. Ophthalmoplegia was also reported [14],[16].

In our study, optic neuritis was recorded in only one eye. It is very rare sequelae of HZO, that may occur simultaneously to the acute vesicular rash or, more frequently, as a postherpetic complication, up to 10 weeks after disease onset [17],[18]. Optic neuritis is characterized by low vision and central visual field defects, with an optic nerve that looks initially normal or edematous, and which progresses to optic atrophy leading to visual loss like in our patient. A good recovery of HZO optic neuritis with systemic acyclovir and steroid has been also reported [18].

The overall visual outcome was relatively good in our series, with 78.4% of eyes having a final BCVA ≥ 20/40, and only 7.8% of affected eyes were with final BCVA < 20/200. Causes of low vision included optic disc atrophy related to optic neuritis, neurotrophic keratopathy, and corneal opacity. Zaal et al. [8] reported a final VA > 20/25 in 90.4% of cases at 6 months follow-up. As well, Gupta et al. [10] reported a final BCVA of 20/40 or better in 90% of immunocompetent subjects younger than 40 years old. In a series of ten healthy children with HZO, final BCVA was 20/20 in 80% of cases [19]. In a prospective study, 56.3% of patients had a VA of 6/6 or better and visual loss was associated to increasing age, positive Hutchinson's sign, absent corneal sensation, corneal epithelial lesions, and uveitis [20].

In this study, a lower rate of PHN (13.3%) was found than those of previous studies [5],[9],[21]. A higher incidence (17.8%) was reported by Ghaznawi et al. [6] in an American series including 112 patients and was statistically significantly higher in the group aged more than 60 years old. As well, in a Pacific region study, PHN was recorded in 20.9% of patients [5], with 64.3% of patients older than 65 years. The main risk factor for PHN is advancing age; other risk factors include severity of acute zoster pain and rash, a painful prodrome, and ocular involvement [1],[22]. It has been also shown that patients with keratitis, conjunctivitis, or uveitis had a higher risk of developing PHN compared with patients who did not have these ocular features [5].

Our study presents some limitations, related to its retrospective design, bias selection, and varying follow-up. We acknowledge that the use of final VA introduces a potential bias because the disease may be recurrent and continue to cause loss of VA beyond the time the outcome is reported. In addition to these limits, a major drawback is the potential bias selection.

## Conclusions

Our study provided epidemiologic and clinical data of HZO in a Tunisian population. AU and keratitis were the most common ocular manifestations. Neurotrophic keratopathy was scarce.

The overall visual outcome is good, with about three quarters of the treated patients maintaining VA of 20/40 or better.

## Abbreviations

AU: anterior uveitis

BCVA: best corrected visual acuity

HZO: herpes zoster ophthalmicus

IOP: intraocular pressure

PHN: postherpetic neuralgia

VA: visual acuity

## Competing interests

The authors declare that they have no competing interests.

## Authors’ contributions

RK, SA, BJ, and AZA have made substantial contributions to the conception and design, or acquisition of data, or analysis and interpretation of data. RK, SA, SZ, SZ, SBY, and MK have been involved in drafting the manuscript or revising it critically for important intellectual content. MK has given the final approval of the version to be published. RK and MK agree to be accountable for all aspects of the work in ensuring that the questions related to the accuracy or integrity of any part of the work are appropriately investigated and resolved. All authors read and approved the final manuscript.

## References

[B1] Liesegang TJ Herpes zoster ophthalmicus natural history, risk factors, clinical presentation, and morbidity Ophthalmology 2008 115 2 Suppl S3 12 10.1016/j.ophtha.2007.10.009 18243930

[B2] Standardization of uveitis nomenclature for reporting clinical data. Results of the first international workshop Am J Ophthalmol 2005 140 509 516 10.1016/j.ajo.2005.03.057 16196117 PMC8935739

[B3] Dworkin RH Schmader RE Treatment and prevention of post herpetic neuralgia Clin Infect Dis 2003 36 877 882 10.1086/368196 12652389

[B4] Cebrián-Cuenca AM Díez-Domingo J San-Martín-Rodríguez M Puig-Barberá J Navarro-Pérez J Epidemiology and cost of herpes zoster and postherpetic neuralgia among patients treated in primary care centres in the Valencian Community of Spain BMC Infect Dis 2011 1 11 302 10.1186/1471-2334-11-302 22044665 PMC3235981

[B5] Borkar DS Tham VM Esterberg E Ray KJ Vinoya AC Parker JV Uchida A Acharya NR Incidence of herpes zoster ophthalmicus: results from the Pacific ocular inflammation study Ophthalmology 2013 120 451 456 10.1016/j.ophtha.2012.09.007 23207173 PMC3594416

[B6] Ghaznawi N Virdi A Dayan A Hammersmith KM Rapuano CJ Laibson PR Cohen EJ Herpes zoster ophthalmicus: comparison of disease in patients 60 years and older versus younger than 60 years Ophthalmology 2011 118 2242 2250 10.1016/j.ophtha.2011.04.002 21788078

[B7] Mullooly JP Riedlinger K Chun C Weinmann S Houston H Incidence of herpes zoster, 1997–2002 Epidemiol Infect 2005 133 245 253 10.1017/S095026880400281X 15816149 PMC2870243

[B8] Zaal MJ Völker-Dieben HJ D’Amaro J Visual prognosis in immunocompetent patients with herpes zoster ophthalmicus Acta Ophthalmol Scand 2003 81 216 220 10.1034/j.1600-0420.2003.00057.x 12780396

[B9] Yawn BP Wollan PC St Sauver JL Butterfield LC Herpes zoster eye complications: rates and trends Mayo Clin Proc 2013 88 562 570 10.1016/j.mayocp.2013.03.014 23664666 PMC3788821

[B10] Gupta N Sachdev R Sinha R Titiyal JS Tandon R Herpes zoster ophthalmicus: disease spectrum in young adults Middle East Afr J Ophthalmol 2011 18 178 182 10.4103/0974-9233.80710 21731332 PMC3119290

[B11] Wang TJ Hu CC Lin HC Increased risk of anterior uveitis following herpes zoster: a nationwide population-based study Arch Ophthalmol 2012 130 451 455 10.1001/archophthalmol.2011.357 22491915

[B12] Liesegang TJ Corneal complications from herpes zoster ophthalmicus Ophthalmology 1985 92 316 324 10.1016/S0161-6420(85)34034-4 3873048

[B13] Park KC Yoon SS Yoon JE Rhee HY A case of herpes zoster ophthalmicus with isolated trochlear nerve involvement J Clin Neurol 2011 7 47 49 10.3988/jcn.2011.7.1.47 21519528 PMC3079161

[B14] Chhabra MS Golnik KC Recovery of ocular motor cranial nerve palsy after herpes zoster ophthalmicus J Neuroophthalmol 2014 34 20 22 10.1097/WNO.0b013e3182a59c69 24051426

[B15] Tsuda H Ito T Yoshioka M Ishihara N Sekine Y Isolated trochlear nerve palsy in herpes zoster ophthalmicus Intern Med 2007 46 535 536 10.2169/internalmedicine.46.6373 17443053

[B16] Hakim W Sherman R Rezk T Pannu K An acute case of herpes zoster ophthalmicus with ophthalmoplegia Case Rep Ophthalmol Med 2012 2012 953910 22649745 10.1155/2012/953910 PMC3357592

[B17] De Mello VB Foureaux EC Porto FB Herpes zoster optic neuritis Int Ophthalmol 2011 31 233 236 10.1007/s10792-011-9443-y 21626168

[B18] Wang AG Liu JH Hsu WM Lee AF Yen MY Optic neuritis in herpes zoster ophthalmicus Jpn J Ophthalmol 2000 44 550 554 10.1016/S0021-5155(00)00204-5 11033135

[B19] De Freitas D Martins EN Adan C Alvarenga LS Pavan-Langston D Herpes zoster ophthalmicus in otherwise healthy children Am J Ophthalmol 2006 142 393 399 10.1016/j.ajo.2006.03.059 16935582

[B20] Nithyanandam S Stephen J Joseph M Dabir S Factors affecting visual outcome in herpes zoster ophthalmicus: a prospective study Clin Experiment Ophthalmol 2010 38 845 850 10.1111/j.1442-9071.2010.02352.x 20572824

[B21] Puri LR Shrestha GB Shah DN Chaudhary M Thakur A Ocular manifestations in herpes zoster ophthalmicus Nepal J Ophthalmol 2011 3 165 171 21876592 10.3126/nepjoph.v3i2.5271

[B22] Opstelten W Mauritz JW de Wit NJ van Wijck AJ Stalman WA van Essen GA Herpes zoster and postherpetic neuralgia: incidence and risk indicators using a general practice research database Fam Pract 2002 19 471 475 10.1093/fampra/19.5.471 12356697

